# Supplementary figures and images for: Potential lineage transmission within the active microbiota of the eggs and the nauplii of the shrimp Litopenaeus stylirostris: possible influence of the rearing water and more
Source: PeerJ. 2021 Nov 15;9:e12241. doi: 10.7717/peerj.12241 (PMC8601056; doi:10.7717/peerj.12241)

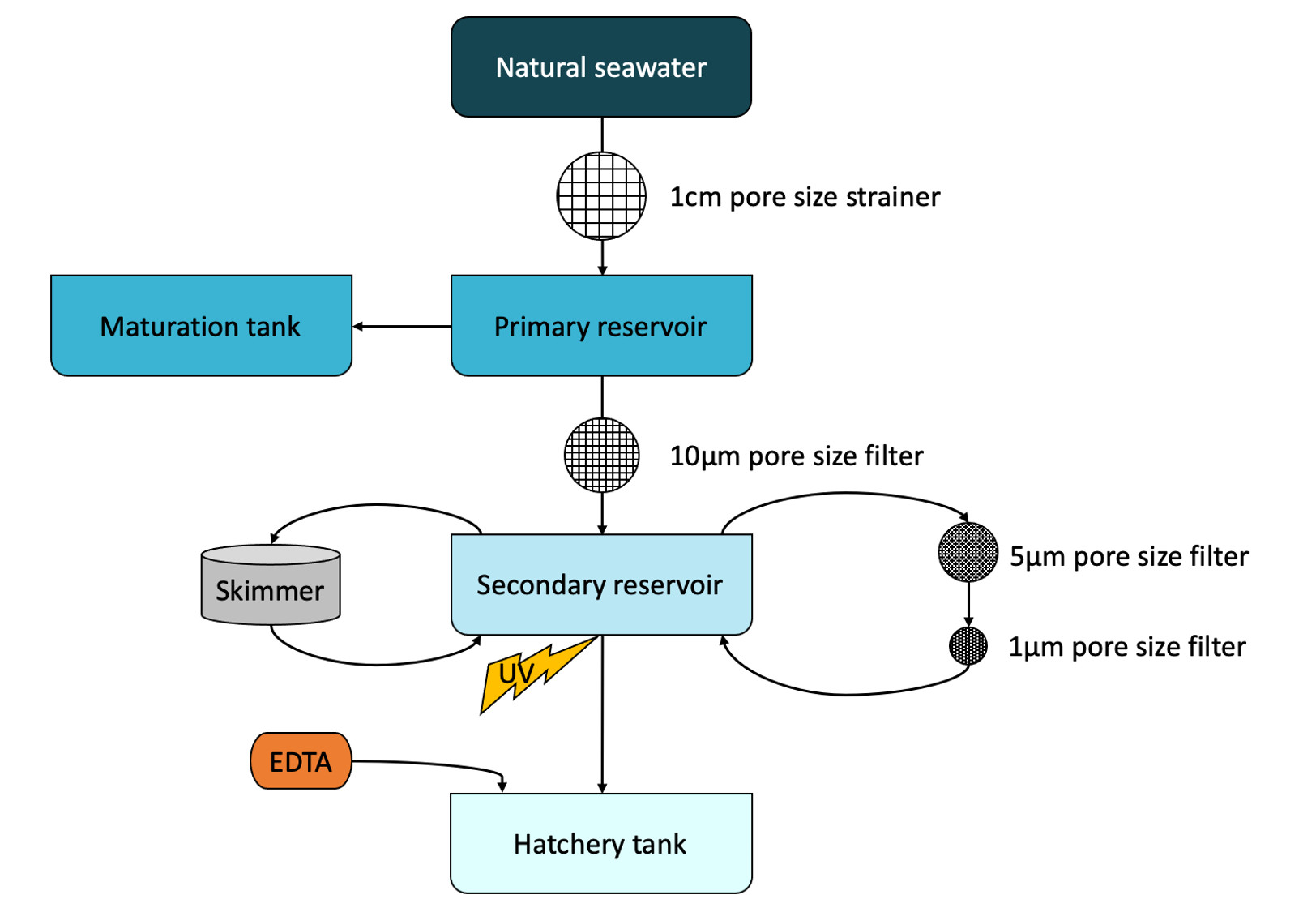

Supplement: Supplemental Information 1 — Natural seawater treatments from the primary reservoir to the secondary reservoirs and the hatchery and maturation tanks. [file peerj-09-12241-s001.jpg]

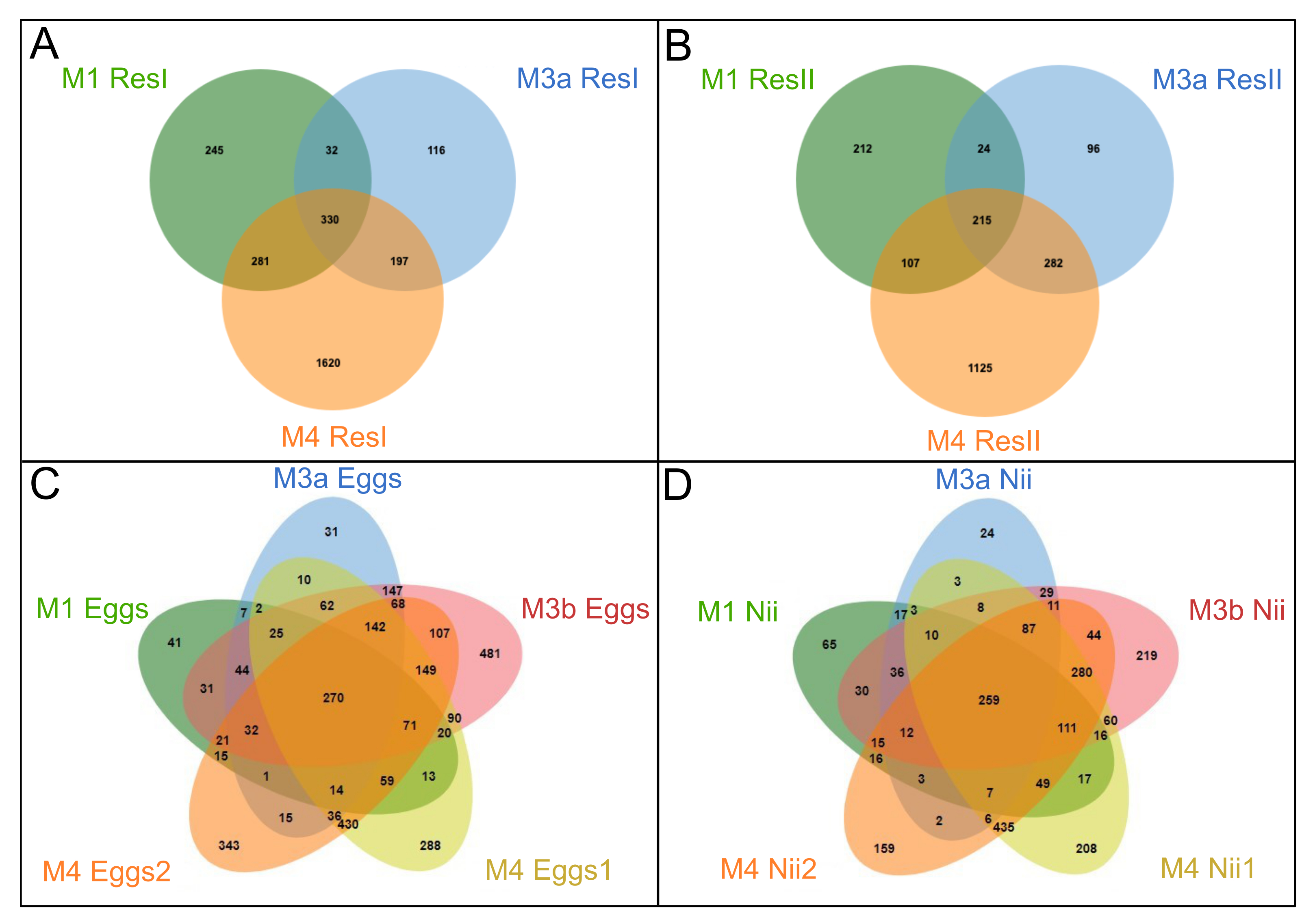

Supplement: Supplemental Information 2 — Venn diagrams of shared and specific ASVs among experiments for A) all the primary reservoir (ResI) samples, B) all the secondary reservoir (ResII) samples, C) all the egg samples and D) all the samples. The green ellipses represent the M1 experiment samples. The blue ellipses represent the M3 experiment samples sequenced during the first sequencing (M3a). The red ellipses represent the M3 experiment samples sequenced during the second sequencing (M3b). The yellow and orange ellipses represent the M4 experiment samples. Numbers noted in the overlapping areas correspond to shared ASVs among samples. Numbers noted outside of the overlapping areas correspond to specific ASVs. [file peerj-09-12241-s002.jpg]

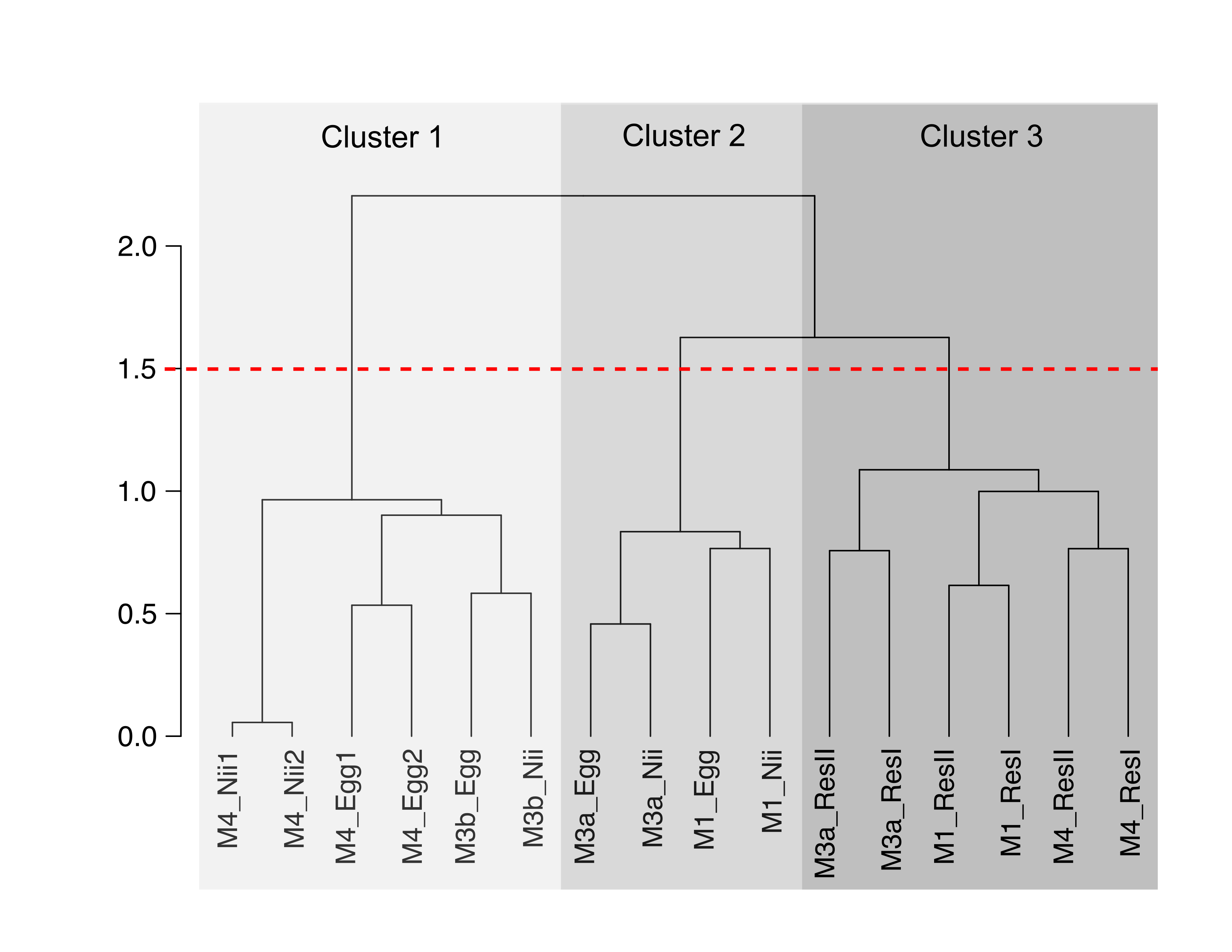

Supplement: Supplemental Information 3 — Hierarchical clustering based on Bray-Curtis dissimilarity and non-normalized data. In order to define clusters, a 1.5 threshold was set (represented by the dotted red line). Cluster 1, in light grey, regroups all the egg and the nauplii (nii) samples from the M3 and M4 experiments and sequenced during the second sequencing (M3b and M4). Cluster 2, in medium grey, regroups the egg and the nauplii (nii) samples from the M1 and M3 experiments and sequenced during the first sequencing (M1 and M3a). Cluster 3, in dark grey, regroups all the water samples (ResI and ResII). [file peerj-09-12241-s003.jpg]
